# Love, laugh, life—the effect of empathy on the processing of emotion-label, emotion-laden and neutral abstract words

**DOI:** 10.1038/s41598-025-18415-x

**Published:** 2025-09-12

**Authors:** Linda Espey, Laura Bechtold, Marta Ghio

**Affiliations:** Institute of Experimental Psychology, Faculty of Mathematics and Natural Sciences, Heinrich Heine University Düsseldorf, Düsseldorf, Germany

**Keywords:** Emotionality, Emotion-label, Emotion-laden, Empathy, Ratings, Lexical decision task, Human behaviour, Psychology

## Abstract

While there is accumulating evidence that emotional words are processed faster than neutral words, findings regarding processing differences between emotion-label and emotion-laden words are inconsistent, potentially due to uncontrolled word- or participant-specific characteristics. We therefore analyzed lexical decision reaction times for emotion-label, emotion-laden, and neutral abstract words, while controlling for subjective differences in word valence, arousal, concreteness, and interoception, as well as considering inter-individual differences in empathy. We neither replicated the facilitatory emotionality effect for emotion-label or emotion-laden compared to neutral word processing nor found evidence for a reaction time difference between emotion-label and -laden words. Notably, however, results showed a word type-specific effect of empathy: Participants reacted faster to specifically emotion-label words, the higher their empathy. Additional exploratory analyses confirmed a word-type-specific gradual pattern, with stronger association of emotion-label than emotion-laden than neutral words with absolute valence, arousal and interoception. These analyses yet again revealed a word-type-specific modulation by empathy, wherein emotion-label words’ ratings were significantly enhanced by empathy. Individual differences in empathy thus seem to affect specifically the processing of emotion-label words and the evaluation of their affective properties. Our findings underline the importance to consider word- and participant-specific characteristics in research on the semantic processing of emotional abstract words.

**Supplementary Information:**

The online version contains supplementary material available at 10.1038/s41598-025-18415-x.

## Introduction

Word processing relies on the retrieval of conceptual representation associated with a word’s form from semantic memory. According to *grounded cognition theory*, conceptual representations comprise multimodal experiential information about the entities a word refers to^1^. We focus here on emotional words, including *emotion-label* words, which refer to discrete emotions (e.g., *anger*,* happiness*), and *emotion-laden* words, which refer to entities that evoke emotions (e.g. *death*,* friendship*)^2^. Psycholinguistic studies across languages suggest that the dominant experiential information that makes up the conceptual representation of emotional words is affective information, namely *valence* (i.e., the extent to which an emotion is positive or negative), and *arousal* (i.e., the emotional intensity)^2–4^. Consistently, emotional compared to neutral word processing elicits greater activation in a distributed affective brain network^2,5^. On the behavioral level, the emotionally-enriched semantic representations of emotional words lead to a faster processing compared to neutral words^6–8^. This processing advantage is referred to as *emotionality effect*.

Most studies investigating the emotionality effect did not distinguish between emotion-label and emotion-laden words, thus partly neglecting the question whether the emotionality effect is driven by emotion-label or emotion-laden words, or both^2^. Among the studies distinguishing between the two emotional word types, most measured reaction times in explicit tasks, e.g., affective categorization, across multiple languages (Arabic^9,10^; Chinese^11–15^; Polish^16^; Spanish^17^. Focusing on studies that used implicit tasks, the emerging pattern of results is controversial. Studies using a primed lexical decision task in English with the words presented in blocks according to the emotional word type^18,19^ found that emotion-label words were processed faster than emotion-laden words. In turn other studies using a Stroop task in Chinese^15^ or a lexical decision task with a randomized presentation order in English^20^ or Chinese^21^ did not find evidence for such a processing advantage for emotion-label compared to emotion-laden words. To our knowledge, no study so far was conducted in German and generalizability across languages has been questioned^16^. Thus, investigating processing differences between German emotion-label vs. emotion-laden words in an implicit task was the first aim of the current study. Yet, this study goes one step further and investigates whether the partially conflicting results might originate from two further potential sources of variance recently requested to be considered in grounded cognition research^22–24^: word- and participant-related characteristics.

While affective information (valence and arousal) seems to similarly characterize the semantic space for emotion-label and emotion-laden words^24,25^, psycholinguistic studies suggest at least two word-related characteristics that might play a role in the differential processing of emotion-label and emotion-laden words. The first characteristic is *concreteness*, i.e., the extent to which a word refers to a tangible, physical referent. Emotion-label words have been shown to be rated as even more abstract than other abstract words^26^, while emotion-laden words can vary along the concreteness dimension (from more concrete, e.g., *bomb*, to more abstract, e.g., *respect*). To rule out that a potential difference between emotion-label and emotion-laden words is driven by a confounding *concreteness effect* (i.e., a processing advantage of concrete over abstract words)^27–31^, we will take into account single word concreteness ratings. The second word characteristic is *interoception*, i.e., the perception of internal bodily states. Interoception has been identified as the dominant modality of perceptual experience for the majority of discrete emotions referred to by emotion-label words^32^. In line with grounded cognition accounts, one could assume that interoceptive information enriches especially the representations of emotion-label words^25^ due to their direct reference to emotions, which might facilitate their processing compared to emotion-laden words. Considering single word interoception ratings is therefore another relevant aspect we would like to consider when testing the fine-grained gradation of the emotionality effect for emotion-label versus emotion-laden words.

Previous research focusing on the psycholinguistic word-related characteristics valence, arousal, concreteness and interoception assessed them in the form of ratings averaged across participants, potentially overlooking individual differences in evaluating such word-specific characteristics. For example, while on average, emotional words are more abstract than other abstract words^26 ^an emotion-label word such as *anger* might be relatively more concrete to a person, who associates it with a red face and stomping footsteps. As we experience concepts in different situations, the grounding of these concepts and underlying semantic properties differ between individuals^33^. Furthermore, not only the specific experience with a concept, but also cultural aspects, as well as imagery and cognitive strategies might lead to differences in the embodiment of concepts^24,34,35^. In line with recent discussions on the importance of individual differences in semantic processing^23,24^ we consider individual ratings for word-related characteristics.

Following the same logic and according to grounded cognition accounts, participant-related characteristics might affect emotional word processing, as individual experience modulates conceptual word meaning representations^33^. The participant-related characteristic that we will consider is *empathy*, as it affects emotional experience and might thus modulate the emotionality effect. Empathy leads to the simulation of the mental or emotional state of another person^36,37^, which is then available for grounding a concept as a source of experiential information. Higher empathy has been shown to lead to a more rapid integration of verbal content and social information^38^. Furthermore, empathy and social skills predicted congruency effects between emotional facial expressions and sentential contexts^39^, and higher versus lower empathy led to slower responses to emotion-laden words after seeing crying faces^40^. When it comes to single word processing, Esteve-Gibert et al.^41^ showed that participants with higher empathy are more sensitive to intonation for disambiguating the meaning of ambiguous words. Silva et al.^42^ found that high compared to low sensitivity for a specific discrete emotion (disgust), an empathy-related concept, was associated with slower reaction times for disgust-related word processing. While this previous research thus suggests that empathy might play a role in grounding the meaning of emotional words, it has not yet been investigated whether empathy modulates the emotionality effect, and if this modulation further differs between emotion-label and emotion-laden words. The grounding of empathetic processes in the emotion processing network^43^ hints at the possibility that empathy might affect the representation and processing of emotion-label words more strongly than that of emotion-laden (and neutral) words.

In the present study, we examined the emotionality effect by further differentiating between emotion-label and emotion-laden words, which were presented in a lexical decision task alongside neutral words, for the first time in German. Importantly, we considered: (i) the word-related characteristics valence, arousal, concreteness and interoception based on ratings collected from the same participants who performed the lexical decision task; (ii) the participants’ empathy as an emotion-related, participant-specific characteristic. To assess the impact of these word- and participant-specific characteristics at the same time, we conducted linear mixed effect (LME) analyses^44,45^ to analyze single-trial reaction times in response to emotion-label, emotion-laden and neutral abstract words in the lexical decision task. We statistically modelled the absolute valence, arousal, concreteness and interoception ratings of each participant as covariates as well as the participants’ individual empathy score.

First, we expected to replicate the facilitatory emotionality effect in the lexical decision task, with faster processing for emotional (emotion-label and emotion-laden) words compared to neutral words. In addition, we expected a more fine-grained reaction time advantage for emotion-label versus emotion-laden words, driven by the richer experiential representation of emotion-label words. This should result in a gradual pattern for the emotionality effect (i.e., faster reaction times for emotion-label than emotion-laden than neutral words). Second, and more importantly, we expected that this gradual pattern for the emotionality effect is all the stronger the higher the participants’ empathy. This should result from a gradual word-type-specific reaction time modulation by empathy (i.e., faster reaction times with higher empathy especially for emotion-label than emotion-laden than neutral words). Notably, while a priori matching of stimuli based on potentially confounding psycholinguistic dimensions is one approach, differences between emotion-label, emotion-laden and abstract neutral words on the respective dimension might vary individually^46^. We thus collected word ratings (on valence, arousal, concreteness and interoception) from the same participants, who performed the lexical decision task, and we control these potentially confounding word-related characteristics in the LME analysis on the reaction times by including them as covariates.

Lastly, in additional exploratory LME analyses of the word ratings as dependent variables, we tested whether empathy modulates the participants’ subjective ratings for valence, arousal, concreteness and interoception differently for emotion-label, emotion-laden and neutral words. We again expected a gradual word-type-specific modulation by empathy, i.e., higher absolute valence, arousal and interoception ratings with higher empathy especially for emotion-label than emotion-laden than neutral words. No modulatory effects of empathy were expected for concreteness ratings.

## Method

### Participants

In total, 55 volunteers participated in the study. We excluded one participant who reported impaired vision and one who reported a history of psychiatric disease. Further, we excluded one participant, whose accuracy in the lexical decision task deviated more than three standard deviations from the sample mean accuracy for the pseudoword condition (indicating a low-compliance response bias). The remaining 52 participants (37 women and 15 men, mean age = 23.7 years, *SD* = 4.9 years, ranging from 18 years to 38 years) were native German speakers, had normal or corrected-to-normal vision, no psychiatric or neurological diseases, no dyslexia and completed at least ten years of school education (*n* = 4) or were students with at least university entrance degree (*n* = 48). The mean empathy score, which was acquired with the German version^47^ of the *Interpersonal Reactivity Index* (IRI)^48^ and included the subscales *Perspective Taking*, *Fantasy* and *Empathic Concern* (potential range: 12 to 60 points), ranged from 35 to 56 points (*M* = 47.0 points, *SD* = 5.4 points). Participants who were psychology students received course credits as compensation. The study was approved by the ethics board of the faculty of Mathematics and Natural Sciences of Heinrich Heine University Düsseldorf and was in accordance with the ethical standard Declaration of Helsinki. All participants provided informed consent prior to participation.

### Material

We selected 120 German words comprising 40 emotion-label, 40 emotion-laden and 40 neutral abstract words. Emotion-label words but not emotion-laden words were classified as ‘feeling’ in GermaNet^49,50^. Further, based on a Spanish data base^51^ of emotional prototypicality ratings collected on a scale from 1 to 5, which included the Spanish translations of 38 out of our 40 German emotion-label words, the mean emotional prototypicality of emotion-label words was 3.93 (*SD* = 0.63, min = 2.65, max = 4.95). For descriptive statistics of the psycholinguistic variables of the selected words, see Table 1. Importantly, in this study we were interested in the absolute valence, namely either positive or negative valence versus neutral, as an experiential dimension that can enrich the semantic representation of emotional words and thus affect their processing^6^. We therefore used the valence ratings on a 9-point Likert scale from − 4 (*very negative*) to 4 (*very positive*) collected from an independent sample of seven German native speakers to select 20 positive and 20 negative words for the pool of emotion-label words, and 20 positive and 20 negative words and for the pool of emotion-laden words. Note that the valence rating for one neutral word was not available due to a technical error. An ANOVA on absolute valence ratings revealed a significant effect of Word Type, *F*(2, 116) = 81.57, *p* < .001. Bonferroni-corrected multiple comparisons revealed the expected significantly higher values for emotion-label than neutral, *p* < .001, and for emotion-laden than neutral words, *p* < .001, but no significant difference between emotion-label and emotion-laden words, *p* = .231. The ANOVA on signed valence ratings revealed that the Word Type effect was not significant, *F*(2, 116) = 0.31, *p* = .736, thus delivering no evidence for a potentially confounding negative or positive bias for emotion-label, emotion-laden or neutral words (for a comparable approach, see Vinson et al.^52^. These pre-experimental valence ratings were an intermediate step to validate our a priori stimulus selection for the lexical decision task. Notably, the psycholinguistic dimensions of interest have been rated again by all participants who did the lexical decision task, and those participant-specific ratings were included in the LME analyses on the lexical decision data (see below). The valence ratings collected from the sample of participants in the lexical decision task confirmed the pattern described above and thus validated our word selection (see Results section below). The three types of words did not differ significantly in word length, *F*(2, 117) = 1.05, *p* = .354, nor in spoken word frequency (taken from the SUBTLEX database)^53^, *F*(2, 117) = 1.17, *p* = .314. For a full list of words see Appendix A in the supplementary material.

**Table 1:** Psycholinguistic characteristics of emotion-label, emotion-laden and neutral words.

Psycholinguistic variable	Word type					
	Emotion-label		Emotion-laden		Neutral	
	*M*	*SD*	*M*	*SD*	*M*	*SD*
|Valence|	2.77	0.58	2.51	0.78	1.02	0.63
Signed Valence	0.03	2.82	-0.13	2.62	0.27	0.83
Length (*n* of letters)	8.98	3.89	9.35	3.50	8.30	2.24
Frequency^1^	27.20	53.86	12.67	33.00	15.15	40.62

Mean (*M*) and standard deviation (*SD*) of psycholinguistic variables. Valence ratings were obtained from a pre-experimental rating on an independent sample of seven German speakers, which was conducted with the aim of selecting the stimuli for the current study. For the descriptive statistics of |valence| ratings obtained in the experimental sample and used in all the analyses, see Table 3.

^1^From SUBTLEX data base^53^.

The 120 words selected as experimental stimuli served as input to the software *Wuggy*^54^ to generate 120 pseudowords with the German language module, which were of the same length, the same sub-syllabic structure and transition frequencies as the input words.

### Procedure

Data acquisition took place online as single subject testing sessions assisted by an experimenter via a video conference (implemented via Cisco Webex). Experimenters followed a standardized protocol to assure compliance and instructional understanding by the participants. In the first part of the study, participants performed the lexical decision task. In addition, we collected the demographic data and administered the German version of the IRI^47^. This first part of the study was programmed in Psychopy (version 3.0, www.psychopy.org)^55^ and executed on the Pavlovia online platform (www.pavlovia.org). The second part of the study comprised the ratings for valence, arousal, concreteness and interoception for the 120 words used as stimuli in the lexical decision task. Importantly these ratings were collected from the same sample of participants who performed the lexical decision task and were later entered as covariates into the LME analyses (see below). The ratings were collected via SoSciSurvey^56^ and executed on www.soscisurvey.com. The whole study took 45 to 60 min.

#### Lexical decision task

**Fig. 1:**
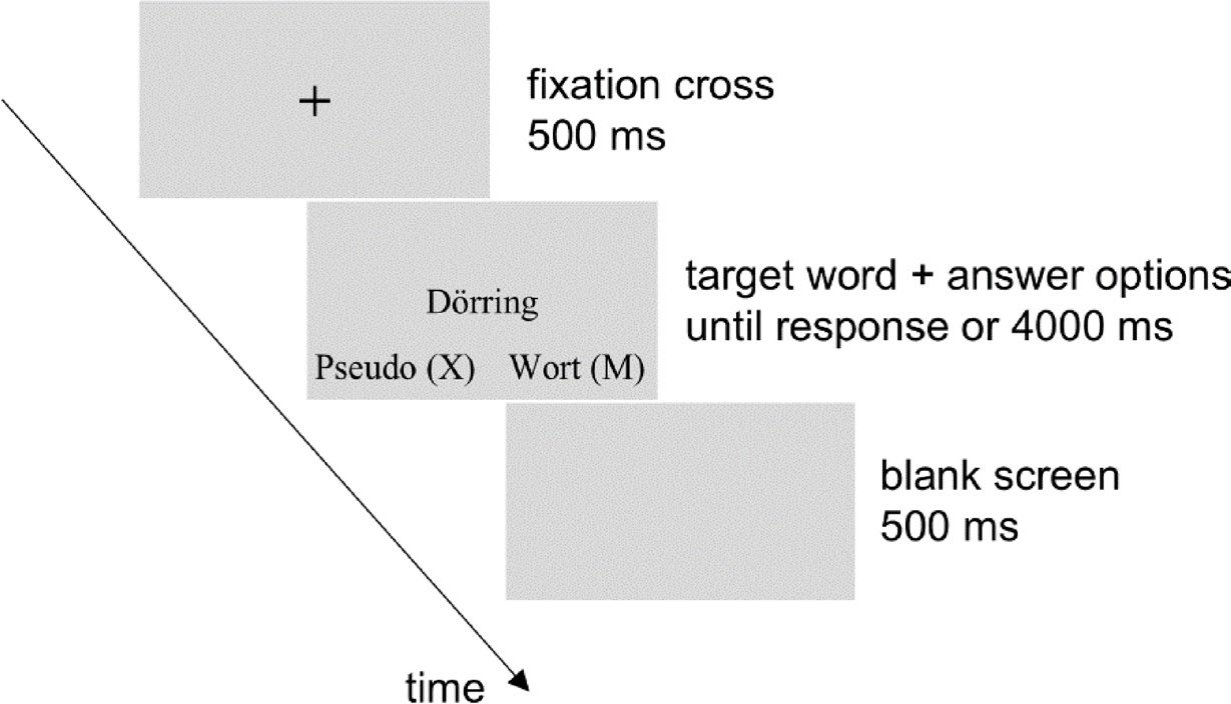
Example of an experimental trial in the lexical decision task.

Participants read the instructions for the lexical decision task and were presented with an example trial to become familiar with the task (the word used in this example was not used as stimulus in the task). An experimental trial of the lexical decision task is displayed in Fig. 1. Each experimental trial started with a fixation cross in the center of the screen displayed for 500 ms, followed by the target, which could be either an emotion-label, an emotion-laden, a neutral word or a pseudoword. At the same time, the answer options, namely ‘pseudo (X)’ and ‘word (M)’ were displayed below the target on the left and right side of the screen, respectively, to remind the participants that they had to make their choice by pressing the left key (‘X’) or the right key (‘M’) to indicate whether the target was, respectively, a pseudoword or a word. Participants were instructed to leave their left index finger on the ‘X’ key and right index finger on the ‘M’ key for the entire duration of the lexical decision task. In our sample of right-handed participants, this meant that responses to words were given with the dominant hand. Participants were instructed to answer as fast and accurately as possible. After their response, an intertrial interval of 500 ms depicting a blank screen was shown before the next trial started. If participants did not respond within 4000 ms, the next trial started automatically. All the 120 words (40 emotion-label, 40 emotion-laden, and 40 neutral words) and the 120 pseudowords were presented once in a random order, resulting in a total of 240 trials. Every 30 trials, participants could take a break of self-administered duration.

#### Ratings

After completing the lexical decision task, participants performed the word ratings. For each scale, participants were provided with detailed instructions and two example words for each extreme of each scale, which were not used as stimuli in the task. First, participants used 9-point Likert scales to rate all experimental words for valence (1 = *very negative*, 5 = *neutral*, 9 = *very positive*) and arousal (9-point Likert scale, 1 = *low*, 9 = *high*; based on Bradley and Lang^57^). Then, they used 5-point Likert scales to rate all experimental words for concreteness (1 = *abstract*, 5 = *concrete*) and interoception (1 = *low association with interoception*, 5 = *high association with interoception*; based on Connell, et al.^32^). For the latter two scales, we presented 40 additional concrete words to make sure the whole scale was covered. For each scale, 25 words were displayed on each page of the online questionnaire. The words on each page were the same across participants but randomized in order, as were the pages and the scales.

### Data analysis

We analyzed the data using R (version 4.2.2) in the RStudio environment (version 2023.03.1). For the LME analyses, we used the R packages lme4 (version 1.1–34)^58^ and lmerTest (version 3.1-3)^59 ^which apply Satterthwaite’s method to estimate degrees of freedom and significance^60^. Interactions were resolved using the R package interactions (version 1.1.5)^61^.

#### Reaction times in the lexical decision task

We verified that every participant reached an overall accuracy higher than 75%. We excluded one positive emotion-label and one negative emotion-laden word from further analysis, as their mean accuracy across all participants was below 75%. We removed pseudowords, trials with missing response (*n* = 2), and incorrect trials (*n* = 176) from further analysis. The average lexical decision task accuracy reached 95.8% (*SD* = 20.0%), ranging from 81.4 to 99.6% per participants. The mean accuracy for emotion-label words was 97.9% (*SD* = 14.2%), for emotion-laden words 97.4% (*SD* = 15.8%), for neutral words 96.1% (*SD* = 19.5%), and for pseudowords 94.5% (*SD* = 22.7%). As the accuracy thus showed ceiling effects, which in turn can mask possible effects of experimental factors (e.g., Katz, et al.^62^), we used accuracy only for excluding incorrect trials as described above, and it was not further analyzed.

For the LME analysis on reaction times in the lexical decision task, we defined a model including the categorical factor Word Type (within-subject, three levels: emotion-label, emotion-laden, neutral). To allow all pairwise comparisons of the three emotionality levels, two contrast matrices were set up: one with neutral as reference condition and one with emotion-label as reference condition. The model also included the mean-centered, continuous factor Empathy as well as the Word Type by Empathy interaction as fixed effects. To control for potential confounding effects and individual differences therein, we modelled as covariates the mean-centered Absolute Valence, Arousal, Concreteness and Interoception based on the word ratings provided by each participant who performed the lexical decision task. For each covariate, we modelled its main effect as well as its interaction with Word Type (see below). As random effects, we included the slope and intercept for Participants and Words. We did not include a random slope of Word Type for the Participants intercept as it produced a singular fit. To sum up, the model was:

$$\begin{aligned} & Reaction{\text{ }}times\,\sim \,Word{\text{ }}Type*Empathy\,+\,Absolute{\text{ }}Valence\, \hfill \\ & +\,Word{\text{ }}Type{\text{ }}:{\text{ }}Absolute{\text{ }}Valence\,+\,Arousal\,+\,Word{\text{ }}Type{\text{ }}:{\text{ }}Arousal\, \hfill \\ & +\,Concreteness\,+\,Word{\text{ }}Type{\text{ }}:{\text{ }}Concreteness\,+\,Interoception\, \hfill \\ & +\,Word{\text{ }}Type{\text{ }}:{\text{ }}Interoception{\text{ }}+{\text{ }}\left( {1|Participant} \right){\text{ }}+{\text{ }}\left( {1|Word} \right) \hfill \\ \end{aligned}$$

We performed an outlier detection based on Cook’s distance^63^ using the R package influence.ME (version 0.9-9)^64^. Values ranged from < 0.01 to 0.07 (*M* = 0.02, *SD* = 0.02). Thus, none of the Cook’s distance values exceed the cut-off of 1 originally suggested by Cook^63^ nor the more conservative cut-off of ~ 0.16 suggested by Jayakumar and Sulthan^65^ (based on simulations for our sample size and number of factors). We then applied an outlier criterion based on model criticism^66^ by excluding trials with standardized residuals higher than 2.5 or lower than − 2.5 (*n* = 151 data points). After exclusions, a total of 5804 data points were included into the LME analysis on the reaction times in the lexical decision task.

We resolved the interaction between our two factors of interest Word Type and Empathy in two ways to fully explore whether the pattern was congruent with our predictions stated in the hypotheses. First, we examined the emotionality effect separately for lower and higher empathic participants. Specifically, we calculated two models, one in which we kept Empathy constant at high levels by shifting the continuous factor Empathy by one standard deviation downward from the mean, and another one in which we kept Empathy constant at low levels by shifting the continuous factor Empathy by one standard deviation upward from the mean. For both models, we tested the effect of Word Type for significance. Second, we tested a word-type-specific modulation by empathy. For this, we computed the planned contrasts testing the effect of Empathy for emotion-label versus neutral, emotion-laden versus neutral, and emotion-label versus emotion-laden words, and we applied a simple slope analysis testing the effect of Empathy for each level of Word Type.

Additionally, we performed two LME analyses on the reaction times with the purpose of testing the effects of absolute Valence and Gender (for details, see Appendix B and C in the supplementary material). These analyses did not address the main experimental question of this study but (i) contributed to the comparison of our results with previous investigations on the emotionality effect that considered the polarity of words (for a review, see Citron, et al.^4^, and (ii) tested the potential confounding effect of Gender^67,68^.

#### Ratings

To test the effect of Word Type and Empathy on the word ratings for each of the assessed rating scales, we conducted LME analyses on the ratings separately for absolute valence, arousal, concreteness and interoception. For all four LME analyses, we defined the same fixed and random effects. Specifically, we modelled the categorical fixed-effect Word Type (three levels: emotion-label, emotion-laden and neutral; contrast matrices as described above). The model also included the mean-centered, continuous fixed-effect factor Empathy as well as its interaction with Word Type. As random-effect factors we included the slope and intercept for Participants and Words. We further included a random slope of Word Type for the Participant intercept. The four LME analyses differed only with respect to the dependent variable, namely either the absolute valence, arousal, concreteness or interoception ratings. To sum up, the model was:

$$Rating\,\sim \,Word{\text{ }}Type*Empathy{\text{ }}+{\text{ }}(1\,+\,Word{\text{ }}Type\left| {Participant){\text{ }}+{\text{ }}(1} \right|Word)$$

Separately for each model, we applied an outlier criterion based on model criticism as described above. For the LME model on absolute valence ratings, 94 trials (1.6%) were excluded so that 5861 data points remained. For the LME on arousal ratings, 50 trials (0.8%) were excluded so that 5905 data points remained. For the LME on concreteness ratings, 96 trials (1.6%) were excluded so that 5859 data points remained. For the LME analysis on interoception ratings, 113 trials (1.9%) were excluded so that 5842 data points remained.

For each model, we resolved the significant interaction of Word Type and Empathy by looking for a word-type-specific rating modulation by Empathy. Specifically, we computed the planned contrasts testing the effect of Empathy for emotion-label versus neutral, emotion-laden versus neutral, and emotion-label versus emotion-laden words, and we applied a simple slope analysis testing the effect of Empathy for each level of Word Type.

## Results

### Reaction times in the lexical decision task

**Fig. 2:**
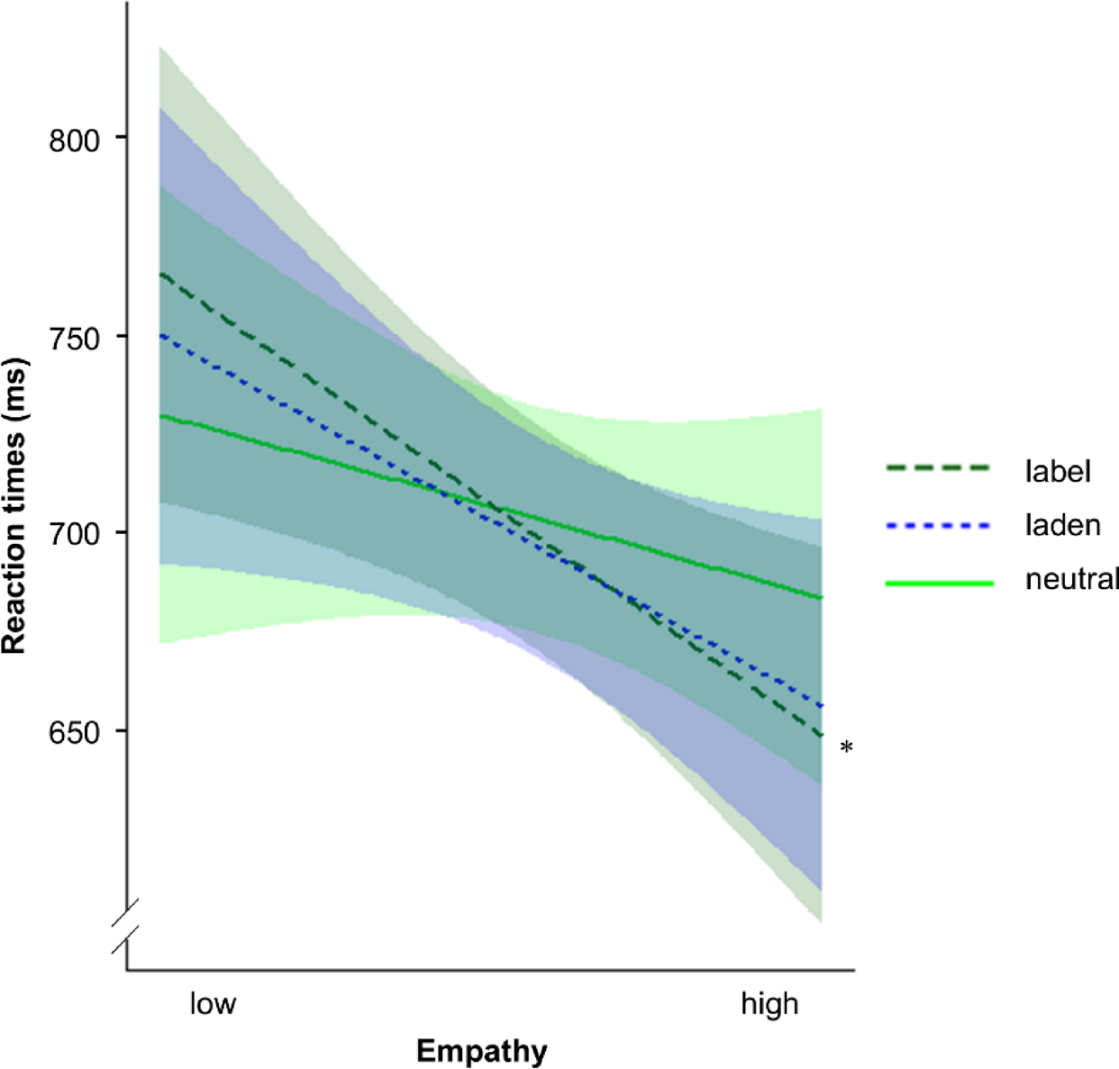
Slope estimates for the effect of Empathy per Word Type on the reaction times in the lexical decision task. Semitransparent ribbons reflect 90% confidence intervals. Label = emotion-label words; laden = emotion-laden words. * *p* < .05.

The LME analysis on reaction times in the lexical decision task revealed a significant Word Type × Empathy interaction, *p* = .004. Resolving this interaction via testing the effect of Word Type by Empathy, we found that the effect of Word Type was neither significant for participants with lower empathy, *p* = .720, nor for participants with higher empathy, *p* = .361. Resolving this interaction additionally via testing the effect of Empathy by Word Type, planned contrasts revealed a significantly stronger effect of Empathy for emotion-label than neutral words, *p* = .001, and for emotion-laden than neutral words, *p* = .026, while there was no significant difference between emotion-label and emotion-laden words, *p* = .288. Post-hoc simple slope analyses revealed that higher Empathy led to significantly faster reaction times in response to emotion-label words, *p* = .035. Descriptively, but not significantly, the same could be observed for emotion-laden words, *p* = .087, and neutral words, *p* = .394. For the slope estimates Empathy per Word Type, see Fig. 2.

Regarding the included rating-based covariates, there was only a significant main effect of Interoception, *p* = .031, with higher interoception ratings leading to faster responses in the lexical decision task. All other main and interaction effects were not significant, all *p*s ≥ .077. For inferential statistics of the LME analysis, see Table 2.

**Table 2:** Inferential statistics of the LME analysis on reaction times in the lexical decision task.

Effect	β-estimate	SE	df	t/F^1^	*p*
Word type			2, 140.6	0.11	.898
Empathy	− 2.20	2.56	55.83	− 0.86	.394
|Valence|	− 1.33	3.69	5729.20	− 0.36	.719
Arousal	0.42	2.04	5745.54	0.21	.838
Concreteness	4.41	2.99	5773.24	1.47	.140
Interoception	− 8.21	3.80	5778.35	− 2.16	.031*
Word type × empathy			2, 5640.6	5.45	.004**
Resolution word type by empathy					
Word type for low empathy			2, 165.3	0.33	.720
Word type for high empathy			2, 190.9	1.02	.361
Resolution empathy by word type					
Planned contrasts					
Empathy for label vs. neutral	− 3.37	1.05	5648.96	− 3.22	.001**
Empathy for laden vs. neutral	− 2.27	1.02	5636.58	− 2.23	.026*
Empathy for label vs. laden	− 1.10	1.04	5636.53	− 1.06	.288
Simple slopes					
Empathy for label	− 5.57	2.57		− 2.17	.035*
Empathy for laden	− 4.46	2.56		− 1.74	.087
Empathy for neutral	− 2.20	2.56		− 0.86	.394
Word type × |valence|			2, 5741.1	2.56	.077
Word type × arousal			2, 5742.5	0.13	.877
Word type × concreteness			2, 5740.2	2.19	.112
Word type × interoception			2, 5725.7	0.67	.513

The sign of the beta estimates shows the direction of effects. LME = linear mixed effect, |Valence| = absolute valence, *SE* = standard error, *df* = degrees of freedom, label = emotion-label words, laden = emotion-laden words. ^1^*t*-statistic for all effects except for main and interaction effects including Word Type. For effects including Word Type, *F*-statistic is reported and beta values are not available. * *p* < .05, ** *p* < .01.

### Ratings

For the descriptive statistics of the psycholinguistic ratings obtained from the experimental sample after the lexical decision task for emotion-label, emotion-laden, and neutral words on valence, arousal, concreteness and interoception, see Table 3.

**Table 3:** Descriptive statistics of the word ratings for emotion-label, emotion-laden and neutral words.

Psycholinguistic variable	Word type					
	Emotion-label		Emotion-laden		Neutral	
	*M*	*SD*	*M*	*SD*	*M*	*SD*
|Valence|	2.66	1.18	2.40	1.31	0.98	1.18
Arousal	6.00	2.55	5.13	2.64	3.69	2.34
Concreteness	2.13	1.24	2.58	1.39	2.82	1.45
Interoception	3.66	1.39	2.71	1.49	1.87	1.26

Mean (*M*) and standard deviation (*SD*) of the psycholinguistic variables rated by the experimental sample (*n* = 52) after the lexical decision task for the emotion-label (*n* = 39), emotion-laden (*n* = 39) and neutral words (*n* = 40) included in the final analyses. Words were rated on valence (9-point Likert scale, 1 = *very negative*, 5 = *neutral*, 9 = *very positive*, transformed to the absolute of the signed valence ratings, from |-4| to |4| [|Valence|]), arousal (9-point Likert scale, 1 = *low*, 9 = *high*), concreteness (5-point Likert scale, 1 = *abstract*, 5 = *concrete*) and interoception (5-point Likert scale, 1 = *low association with interoception*, 5 = *high association with interoception*).

**Table 4:** Inferential statistics of the LME analysis on the psycholinguistic word ratings.

Effect	β-estimate	SE	df	t/F^1^	*p*
A. Absolute valence					
Word type			2, 124.91	80.58	< .001***
Planned contrasts					
Label vs. neutral	1.79	0.14	131.60	12.36	< .001***
Laden vs. neutral	1.55	0.14	132.36	10.73	< .001***
Label vs. laden	0.24	0.11	129.57	2.17	.032*
Empathy	< 0.01	0.02	49.73	0.15	.884
Word type × empathy			2, 49.72	4.11	.022*
Planned contrasts					
Empathy for label vs. neutral	0.05	0.02	49.79	2.39	.021*
Empathy for laden vs. neutral	0.03	0.02	49.79	1.49	.142
Empathy for label vs. laden	0.02	0.01	49.63	2.11	.040*
Simple slopes					
Empathy for label	0.05	0.02		3.02	.004**
Empathy for laden	0.03	0.02		2.04	.047*
Empathy for neutral	< 0.01	0.02		0.15	.884
B. Arousal					
Word type			2, 116.43	27.69	< .001***
Planned contrasts					
Label vs. neutral	2.35	0.32	105.09	7.37	< .001***
Laden vs. neutral	1.51	0.25	139.86	5.93	< .001***
Label vs. laden	0.84	0.23	142.52	3.65	< .001***
Empathy	− 0.09	0.04	49.93	− 2.24	.030*
Word type × empathy			2, 50.22	6.80	.002**
Planned contrasts					
Empathy for label vs. neutral	0.17	0.05	49.99	3.62	< .001***
Empathy for laden vs. neutral	0.11	0.03	49.93	3.59	< .001***
Empathy for label vs. laden	0.06	0.02	49.76	2.60	.012*
Simple slopes					
Empathy for label	0.08	0.03		2.49	.016*
Empathy for laden	0.02	0.03		0.71	.479
Empathy for neutral	− 0.09	0.04		− 2.24	.030*
C. Concreteness					
Word type			2, 116.85	10.10	< .001***
Planned contrasts					
Label vs. neutral	− 0.73	0.17	100.45	− 4.28	< .001***
Laden vs. neutral	− 0.26	0.13	140.37	− 1.97	.051
Label vs. laden	− 0.47	0.12	146.24	− 3.81	< .001***
Empathy	0.01	0.02	49.96	0.31	.755
Word type × empathy			2, 49.69	3.27	.046*
Planned contrasts					
Empathy for label vs. neutral	− 0.04	0.03	49.89	− 1.50	.141
Empathy for laden vs. neutral	− 0.01	0.02	49.61	− 0.41	.681
Empathy for label vs. laden	− 0.03	0.01	50.14	− 2.37	.022*
Simple slopes					
Empathy for label	− 0.03	0.02		− 1.27	.209
Empathy for laden	< 0.01	0.02		0.01	.989
Empathy for neutral	0.01	0.02		0.31	.755
D. Interoception					
Word type			2, 117.46	53.78	< .001***
Planned contrasts					
Label vs. neutral	1.83	0.18	91.43	10.37	< .001***
Laden vs. neutral	0.87	0.13	137.42	6.67	< .001***
Label vs. laden	0.96	0.14	129.42	6.99	< .001***
Empathy	− 0.01	0.02	49.92	− 0.55	.587
Word type × empathy			2, 49.99	3.42	.040*
Planned contrasts					
Empathy for label vs. neutral	0.07	0.03	49.95	2.59	.013*
Empathy for laden vs. neutral	0.03	0.02	49.95	1.72	.092
Empathy for label vs. laden	0.04	0.02	49.95	2.35	.023*
Simple slopes					
Empathy for label	0.06	0.03		2.19	.033*
Empathy for laden	0.02	0.03		0.66	.512
Empathy for neutral	− 0.01	0.02		− 0.55	.587

LME = Linear Mixed Effect, *SE* = standard error, *df* = degrees of freedom, label = emotion-label words, laden = emotion-laden words. The sign of the beta estimates shows the direction of effects. ^1^*t*-statistic for all effects except for main and interaction effects including Word Type. For effects including Word Type, *F*-statistic is reported and beta values are not available. * *p* < .05, ** p < .01, *** *p* < .001.

#### Valence

**Fig. 3:**
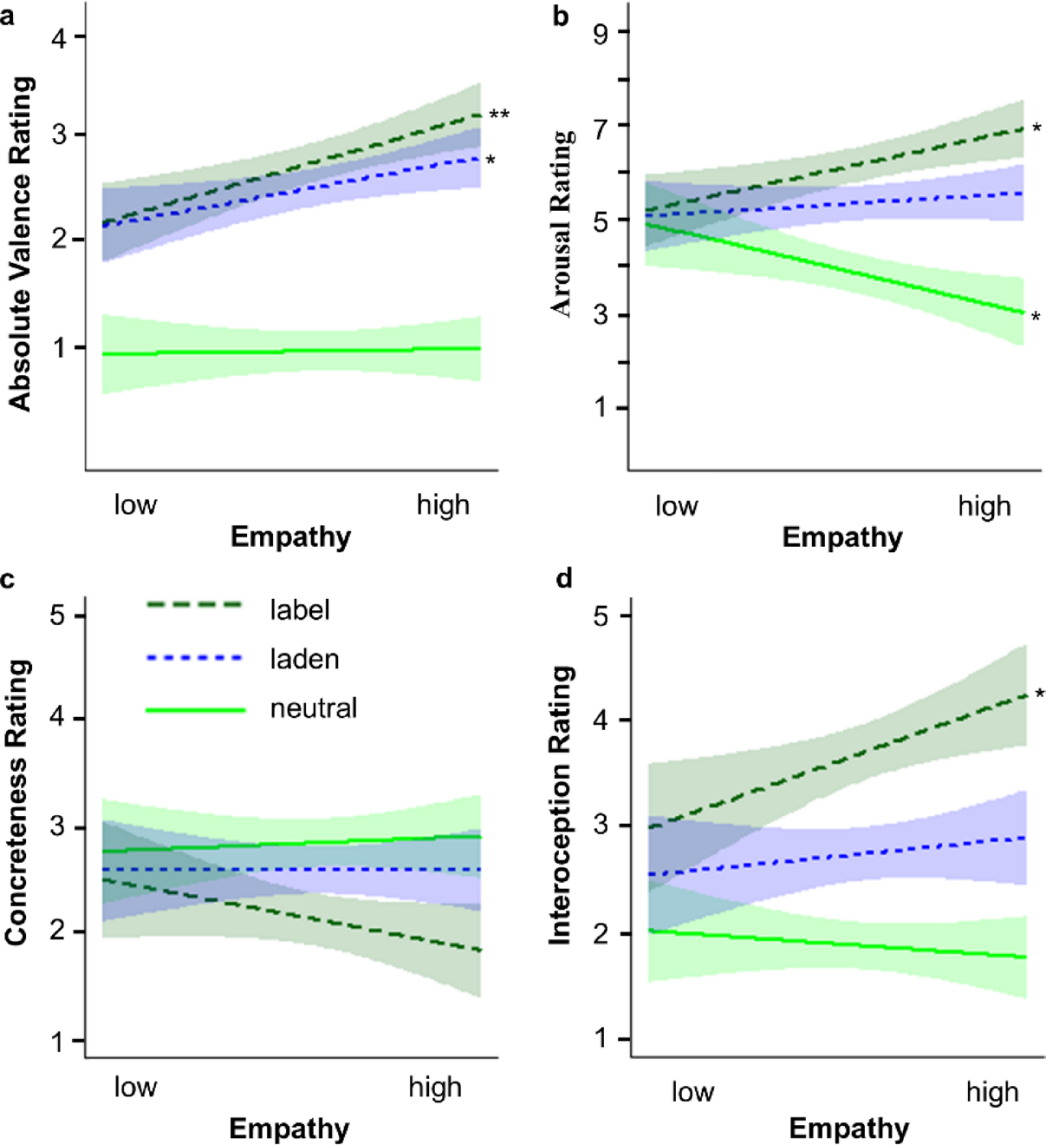
Slope estimates for the effect of Empathy per Word Type on the psycholinguistic ratings. (**a**) absolute valence ratings (possible values from 0 to 4), (**b**) arousal ratings (possible values from 1 to 9), (**c**) concreteness ratings (possible values from 1 to 5), and (**d**) interoception ratings (possible values from 1 to 5). Semitransparent ribbons reflect 90% confidence intervals. Label = emotion-label words; laden = emotion-laden words. *p < .05, **p < .01.

The LME analysis on absolute valence ratings revealed a significant main effect of Word Type, *p* < .001. Planned contrasts revealed that both emotion-label and emotion-laden words received significantly higher ratings than neutral words, both *p*s < .001, and that emotion-label words received significantly higher ratings than emotion-laden words, *p* = .032. There was also a significant interaction between Word Type and Empathy, *p* = .022. Planned contrasts revealed a significantly stronger effect of empathy on absolute valence ratings for emotion-label than neutral words, *p* = .021 and for emotion-label than emotion-laden words, *p* = .040, while there was no significant difference between emotion-laden and neutral words, *p* = .142. Post-hoc simple slope analyses showed that higher Empathy led to significantly higher absolute valence ratings for emotion-label words, *p* = .004, and for emotion-laden words, *p* = .047, while Empathy did not significantly affect absolute valence ratings of neutral words, *p* = .884. For the slope estimates see Fig. 3a. For inferential statistics, see Table 4A.

#### Arousal

The LME analysis on arousal ratings revealed a significant main effect of Word Type, *p* < .001. Planned contrasts revealed that both emotion-label and emotion-laden words received significantly higher arousal ratings than neutral words, and that emotion-label words received higher ratings than emotion-laden words, all *p*s < .001. We also found a significant effect of Empathy, *p* = .030, and a significant Word Type × Empathy interaction, *p* = .002. Planned contrasts revealed that the effect of Empathy was significantly stronger for emotion-label than neutral words, *p* < .001, emotion-laden than neutral words, *p* < .001, and emotion-label than emotion-laden words, *p* = .012. Post-hoc simple slope analyses revealed that higher Empathy led to significantly higher arousal ratings for emotion-label words, *p* = .016, and to significantly lower arousal ratings for neutral words, *p* = .030, while there was no significant modulation for emotion-laden words, *p* = .479. For the slope estimates see Fig. 3b. For inferential statistics, see Table 4B.

#### Concreteness

The LME analysis on concreteness ratings revealed a significant main effect of Word Type, *p* < .001. Planned contrasts showed that emotion-label words received significantly lower concreteness ratings than emotion-laden, *p* < .001, and neutral words, *p* < .001, while emotion-laden and neutral words did not differ significantly, *p* = .051. Further, the analysis revealed a significant Word Type × Empathy interaction, *p* = .046. Planned contrasts revealed that the effect of Empathy was significantly stronger for emotion-label than emotion-laden words, *p* = .022, and did not differ significantly for emotion-label versus neutral or emotion-laden versus neutral words, both *p*s ≥ .141. Post-hoc simple slope analyses revealed that Empathy did not affect concreteness ratings significantly for any level of Word Type, all *p*s ≥ .209. For the slope estimates see Fig. 3c. For inferential statistics, see Table 4C.

#### Interoception

The LME analysis on interoception ratings revealed a significant main effect of Word Type, *p* < .001. Planned contrasts revealed that interoception ratings were significantly higher for emotion-label than neutral, emotion-laden than neutral and emotion-label than emotion-laden words, all *p*s < .001. Further, we found a significant interaction between Word Type and Empathy, *p* = .040. Planned contrasts revealed that the effect of Empathy was significantly stronger for emotion-label than neutral words, *p* = .013, and emotion-label than emotion-laden words, *p* = .023, while emotion-laden and neutral words did not differ significantly, *p* = .092. Post-hoc simple slope analyses revealed that higher Empathy led to significantly higher interoception ratings only for emotion-label words, *p* = .033, and neither for emotion-laden nor neutral words, both *p*s ≥ .512. For the slope estimates see Fig. 3d. For inferential statistics, see Table 4D.

## Discussion

In the present study, we examined the processing differences between emotion-label, emotion-laden, and neutral abstract words and a modulation thereof by empathy via LME analyses of single-trial reaction times in a lexical decision task. At the same time, we statistically controlled for individually rated word-specific valence, arousal, concreteness and interoception. We neither replicated the expected facilitatory emotionality effect for emotional (emotion-label or emotion-laden) compared to neutral words nor found evidence for a more fine-grained reaction time difference between emotion-label and emotion-laden words. However, our results revealed the expected word type-specific reaction time modulation by empathy. Specifically, the simple slope analysis revealed that participants reacted significantly faster to emotion-label words the higher their empathy, while there was no evidence of such a modulation for emotion-laden nor neutral words. Partially confirming the expected gradual pattern, contrasts showed that this empathy-driven reaction time modulation was stronger for emotion-label and emotion-laden than neutral words, while we did not find the expected evidence for a difference between emotion-label and emotion-laden words.

Exploratory analyses of the word ratings revealed a gradual word-type-specific association with absolute valence, arousal and interoception resulting from higher values for emotion-label than emotion-laden than neutral words. The effect of empathy on word ratings yet again partially confirmed the expected gradual word-type-specific modulation by empathy: higher empathy led to consistently higher absolute valence, arousal, and interoception ratings for emotion-label words and this modulation was stronger than for neutral words as well as – although restricted to arousal and interoception – for emotion-laden words. Emotion-laden words received higher arousal ratings than neutral words but there was no evidence for such a difference for absolute valence or interoception.

Our results thus do not replicate the facilitatory emotionality effect on reaction times in contrast with behavioral findings of a processing advantage for emotional over neutral words in lexical decision tasks^6,7^ (for a review, see also Conca, et al.^2^. Findings of a more fine-grained reaction time difference between emotion-label and emotion-laden words are less consistent (see, e.g., Zheng, et al.^21^). Our results are in line with Martin and Altarriba^20 ^who also found no evidence for such a difference with a comparable paradigm, namely a lexical decision task with an intermixed presentation of the word types. In contrast, reaction time differences between emotion-label and emotion-laden words have been reported in studies applying a primed lexical decision task with a blocked presentation of the different word types^18,19^. Experimental design- and task-dependence might thus be a viable post-hoc explanation for our null-finding regarding emotion-label versus emotion-laden word processing differences.

However, taken together with the null-finding regarding the more consistently reported emotional versus neutral word processing advantage, a common cause like word- or participant-related characteristics seems a more likely post-hoc explanation. Concerning word-related characteristics, ratings revealed a gradual word-type-specific association with absolute valence, arousal and interoception, with stronger associations for emotion-label than emotion-laden than neutral words. Notably, we controlled for potentially confounding effects of these word-related characteristics in the LME analysis, which might indeed have driven reaction time differences between emotional and neutral words in previous studies^2^. Our rigorous statistical control of the rated variables, however, might have limited the comparability of our results with previous findings. To address this, we conducted an additional exploratory analysis of our reaction time data excluding the rating-based covariates valence, arousal and interoception (reported in Appendix D in the supplementary material). This analysis revealed a comparable inferential pattern as described in the results section above, with the crucial difference of a significant emotion-label versus neutral word processing advantage in participants with higher empathy. Thus, while the rated affective properties might explain the emotionality effect to a certain degree, not controlling them still did not yield an emotionality effect for emotion-laden words nor a processing difference between emotion-label and emotion-laden words.

Another word-related characteristic that could potentially have confounded the emotionality effect in previous studies is concreteness, as there is evidence that concrete words are processed faster compared to abstract words (for reviews, see e.g. Hoffman^28^, Schwanenflugel^69^). In our study, controlling for a potential concreteness effect on reaction times was crucial as, although we selected only abstract emotional and neutral words (based on an independent rating), our participants still rated emotion-label words significantly lower in concreteness than emotion-laden and neutral words, and the same was descriptively observed for emotion-laden versus neutral words. Notably, the additional analyses on signed valence (see Appendix B in the supplementary material) and excluding the emotional covariates valence, arousal and interoception (see Appendix D in the supplementary material) showed a significant interaction of Concreteness and Word Type as well, which resulted from emotion-label words with higher concreteness being processed significantly slower than those with lower concreteness ratings, underlining the importance to control this confounding variable.

Interoception was the only covariate included in the main LME analysis that significantly affected the reaction times, with words with higher subjective interoception ratings being processed faster than words with lower interoception ratings, irrespective of the specific word type. This is in line with a study^32^ showing that interoceptive information serves as a perceptual modality for grounding abstract concepts and enriches their conceptual representation, thus leading to a processing advantage. It is also consistent with the more general hypothesis that different forms of semantic richness can lead to a processing advantage, and that the greater the semantic richness the greater is the resulting processing advantage^70–72^.

Regarding participant-related characteristics, we found evidence for the expected word-type-specific reaction time modulation by empathy. While this might have added variance, which potentially covered up reaction time differences between the word types (see Fig. 2), it more importantly hints at a more complex manifestation of the grounding of emotional word processing in interplay with empathy. Although the reaction time advantage driven by empathy was only significant for emotion-label words, the contrasts revealed that it was larger for emotion-label as well as for emotion-laden than neutral words and the beta estimates were descriptively in line with the expected gradual effect. Importantly, the additional analyses reported in the supplementary material (i) including signed valence, Appendix B; ii) including gender, Appendix C; and iii) excluding the emotional covariates, Appendix D) draw a consistent picture and support the robustness of the word-type specific modulation by empathy. To our knowledge, no study so far investigated the effect of empathy on emotion-label, emotion-laden and neutral word processing. However, our results are indirectly in line with studies showing a modulatory effect of empathy on contextualized verbal processing^38,39,41^ as well as discrete emotion processing^42^.

A potential explanation for the effect of empathy on the representation and processing of emotion-label compared to emotion-laden and neutral words is the grounding of empathetic processes in the emotion processing network^43^. Furthermore, in accordance with grounded cognition theory, this word-type specific modulation by empathy might be caused by different experiences with emotional stimuli for participants with higher versus lower empathy. This yields the potential explanation for our finding that individuals with higher empathy might simulate emotional states more often than those with lower empathy, which might have resulted in richer experiential information underlying emotional concepts for participants with high empathy scores, ultimately leading to a processing advantage^73,74^. This probably specifically applied to emotion-label words as they refer to discrete emotions^2,75^ and might therefore evoke more emotion-specific simulations, including for example interoceptive experience^25^. While based on the non-significant finding we cannot rule out a comparable (but relatively weaker) mechanism for emotion-laden words, their indirect reference to emotions might have introduced more inter-individual variance in the simulated emotionality, thus reducing the effect of empathy on their processing. Potentially, the processing speed measured in our study was not sensitive to such fine-grained modulations.

The exploratory rating analyses support a word-type-specific modulation by empathy for emotion-label words: The higher the empathy, the higher participants rated emotion-label words in absolute valence, arousal and interoception, while this was the case for emotion-laden words only for absolute valence. This supports the idea that with higher empathy emotion-label words’ representations might be emotionally enriched compared to emotion-laden words, potentially due to stronger reliance on emotional dimensions during simulations in more versus less empathic participants^36,37^.

While this is the first study comparing the processing of German emotion-label versus emotion-laden words in an implicit task and also accounting for participant- and item-specific characteristics in this comparison, thereby adding valuable novel insights, the generalizability of our interpretations is potentially limited. Above, we addressed task-dependence of processing differences between emotion-label, emotion-laden and neutral abstract words, which is supported by previous studies directly comparing different tasks^15,21,76^. Further, there is evidence for language-specific differences, which have been related to characteristics of the respective mental lexica^16^. Future research should thus comprehensively investigate the generalizability vs. specificity of the emotional word type effect across tasks and languages, linking them to specific task demands and language characteristics.

Additionally, null-findings based on our behavioral reaction time measure cannot rule out neural processing differences, e.g., at temporally distinct word processing stages^77–79^, or in the underlying brain areas or networks^2^. There is a growing body of research exploiting the high temporal resolution of electroencephalography to identify distinct processing stages displaying differences between emotion-label and emotion-laden words ^11,13,77,79,80^. These ERP studies however, applied manifold methodological approaches (ranging from, e.g., retrieval processes in a single-word lexical decision task^13,77^ to attentional processes in a dot-probe task^80^ and most of them seem drastically underpowered, as they relied on small sample sizes (e.g., *n* = 15 ^77^ or *n* = 23 in^79^, compare^81^). Future research could look into the brain regions (or their functional connectivity) involved in emotion-label versus emotion-laden word processing via functional magnetic resonance imaging, building on first evidence that emotion-label words might engage a more wide-spread affective brain network^2^. The provided spatial resolution of functional magnetic resonance imaging is of special interest for future research regarding empathy-driven interindividual differences in emotional word processing, given that empathy and emotionality processing seem to rely on partially overlapping neural resources^43,79^.

To conclude, although we could neither replicate the emotionality effect for emotion-label nor for emotion-laden words compared to neutral words in the whole sample nor selectively in participants with high (or low) empathy, we observed a facilitatory effect of higher empathy specifically on emotion-label word processing. This pattern highlights the importance to consider inter-individual characteristics, which are central for the experiential information underlying word meaning representation such as empathy for (direct) emotional experience, when investigating grounded word processing differences^33^. Furthermore, empathy also modulated the individual ratings of word-related characteristics, supporting the idea that processing differences are caused by differences in experiential representational content. In this sense, this study brought evidence for the more recent developments of research on conceptual representations that highlight the importance of considering not only word-related but also participant-related characteristics in examining semantic representation and processing.

## Supplementary Information

Below is the link to the electronic supplementary material.

Supplementary Material 1

## Data Availability

Supporting data and analysis scripts available on OSF: https://osf.io/gveqm/.
