# Supplementary material for: Love, laugh, life—the effect of empathy on the processing of emotion-label, emotion-laden and neutral abstract words
Source: Sci Rep. 2025 Sep 12;15:32468. doi: 10.1038/s41598-025-18415-x (PMC12432158; doi:10.1038/s41598-025-18415-x)
Supplement: Supplementary file 1 — Supplementary Material 1 [file 41598_2025_18415_MOESM1_ESM.pdf]

Supplementary Material

For the manuscript

Love, Laugh, Life – The effect of empathy on the processing of emotion-label, emotion-laden and neutral abstract words

Linda Espey[1], Laura Bechtold[1,2], Marta Ghio[1,2]*

[1]Faculty of Mathematics and Natural Sciences, Heinrich Heine University Düsseldorf, Düsseldorf, Germany

[2]shared last author

Appendix A

List of experimental stimuli

Table A1

Emotion-label, emotion-laden and neutral German words used as stimuli in the lexical decision task and in the psycholinguistic ratings

Emotion-label words	Emotion-laden words	Neutral words
Ärger	Abfall	Absicht
Aufregung	Attraktivität	Algebra
Begeisterung	Aufstieg	Algorithmus
Dankbarkeit	Befreiung	Analysis
Eifersucht	Belastung	Anliegen
Einsamkeit	Betrug	Anstand
Ekstase	Bösartigkeit	Diskretion
Enthusiasmus	Errungenschaft	Division
Enttäuschung	Faschismus	Effekt
Euphorie	Fehlverhalten	Eindruck
Freude	Fertigstellung	Ewigkeit
Frustration	Freundschaft	Gedächtnis
Furcht	Gastfreundschaft	Geometrie
Glück	Glanz	Gewohnheit
Hass	Gleichberechtigung	Grund
Heimweh	Gnade	Koeffizient
Hoffnung	Grausamkeit	Kriterium
Hoffnungslosigkeit	Himmel	Leistung
Liebeskummer	Höflichkeit	Mathematik
Lust	Hölle	Matrix
Missfallen	Ignoranz	Maximum
Mitgefühl	Inzest	Meinung
Mut	Kreation	Neutralität
Neid	Kreativität	Normalität
Nervenkitzel	Misserfolg	Objektivität
Niedergeschlagenheit	Nachteil	Potenz
Scham	Rückgang	Primzahl
Sorge	Sauberkeit	Profit
Stolz	Schnäppchen	Prozent
Sympathie	Schwierigkeit	Reflexion
Traurigkeit	Segen	Regelmäßigkeit
Unbehagen	Skandal	Schema
Unglück	Tücke	Statistik
Unzufriedenheit	Unruhe	Teiler
Vergnügen	Unterdrückung	Term
Verliebtheit	Untreue	Tugend
Wut	Verrat	Vektor
Zärtlichkeit	Verständnis	Vermächtnis
Zufriedenheit	Verwandtschaft	Vorsicht
Zuneigung	Wert	Zurückhaltung

Appendix B

Analysis Including Signed Valence

Method

We conducted an LME analysis on reaction times in the lexical decision task to test the effect of signed valence. We defined a model including the categorical factor Word Type (within-subject, three levels: emotion-label, emotion-laden, neutral). To allow all three pairwise comparisons of the three word types, two contrast matrices were set up: one with neutral words as reference condition and one with emotion-label words as reference condition. The model also included the mean-centered, continuous factor Empathy as well as its interaction with Word Type as fixed effects. To control for potential confounding effects and individual differences therein, we modelled mean-centered Signed Valence, Arousal, Concreteness and Interoception based on the ratings provided by each participant for each word as covariates. For each covariate, we modelled its main effect as well as its interaction with Word Type (see below). As random effects, we included the slope and intercept for Participants and Words. We did not include a random slope of Word Type for the Participants intercept as it produced a singular fit. To sum up, the model was:

*Reaction times ~ Word Type*Empathy + Signed Valence + Word Type:Signed Valence + Arousal + Word Type:Arousal + Concreteness + Word Type:Concreteness + Interoception + Word Type:Interoception + (1|Participant) + (1|Word)*

We performed an outlier detection based on Cook's distance [1] using the R package influence.ME (version 0.9-9) [2]. Values ranged from < 0.01 to 0.06 (M = 0.01, SD = 0.01). Thus, none of the Cook's distance values exceed the cut-off of 1 originally suggested by Cook [1] nor the more conservative cut-off of ~ 0.16 suggested by Jayakumar and Sulthan [3] (based on simulations for our sample size and number of factors). We then applied an outlier criterion based on model criticism [4] by excluding trials with standardized residuals higher than 2.5 or

lower than -2.5 (n = 152 data points). After exclusions, a total of 5803 data points were included into the LME analysis on the reaction times in the lexical decision task.

Results

A detailed summary of the inferential statistics for this additional LME analysis on reaction times in the lexical decision task is reported in Table B1. This analysis revealed a pattern of results consistent with the main analysis reported in the manuscript. We found a significant Word Type × Empathy interaction, $p < .001$. Resolving this interaction via testing the effect of Word Type by Empathy, we found that the effect of Word Type was neither significant for participants with lower empathy, $p = .902$, nor for participants with higher empathy, $p = .125$. Resolving this interaction additionally via testing the effect of Empathy by Word Type, planned contrasts revealed a significantly stronger effect of Empathy for emotion-label than neutral words, $p = .001$, and for emotion-laden than neutral words, $p = .019$, while there was no significant difference between emotion-label and emotion-laden words, $p = .133$. Post-hoc simple slope analyses revealed that higher Empathy led to significantly faster reaction times in response to emotion-label words, $p = .025$. Descriptively, but not significantly, the same could be observed for emotion-laden words, $p = .094$, and neutral words, $p = .446$.

As in the main analysis reported in the manuscript, there was a significant main effect of Interoception, $p = .045$, with higher interoception ratings leading to faster responses in the lexical decision task. Differently from the main analysis, however, this additional analysis revealed a significant Word Type × Concreteness interaction, $p = .035$. Resolving this interaction via testing the effect of Concreteness by Word Type, planned contrasts revealed a significantly stronger effect of Concreteness for emotion-label than emotion-laden words, $p = .011$, while there was neither a significant difference between emotion-label and neutral words, $p = .320$, nor between emotion-laden and neutral words, $p = .106$. Post-hoc simple slope analyses revealed that higher concreteness ratings led to significantly faster reaction

times in response to emotion-label words, $p = .011$, while no significant effect could be observed for emotion-laden, $p = .511$, or neutral words, $p = .128$. All other main and interaction effects were not significant, all ps $\geq .127$. For the slope estimates of Concreteness per Word Type, see Figure B1.

Concerning the effects additionally tested in this analysis, the main effect of Signed Valence was significant, $p = .022$, as well as the Word Type × Signed Valence interaction, $p = .031$. Resolving this interaction via testing the effect of Signed Valence by Word Type, planned contrasts revealed a significantly stronger effect of Signed Valence for emotion-laden than neutral words, $p = .008$, while there was neither a significant difference between emotion-label and neutral words, $p = .134$, nor between emotion-label and emotion-laden words, $p = .210$. Post-hoc simple slope analyses revealed that more positive valence ratings led to significantly faster reaction times in response to neutral words, $p = .022$, while no significant effect could be observed for emotion-label, $p = .660$, or emotion-laden words, $p = .186$. For the slope estimates of Signed Valence per Word Type, see Figure B2.

Figure B1

Slope estimates for the effect of Concreteness per Word Type on the reaction times in the lexical decision task in the LME analysis including the factor Signed Valence

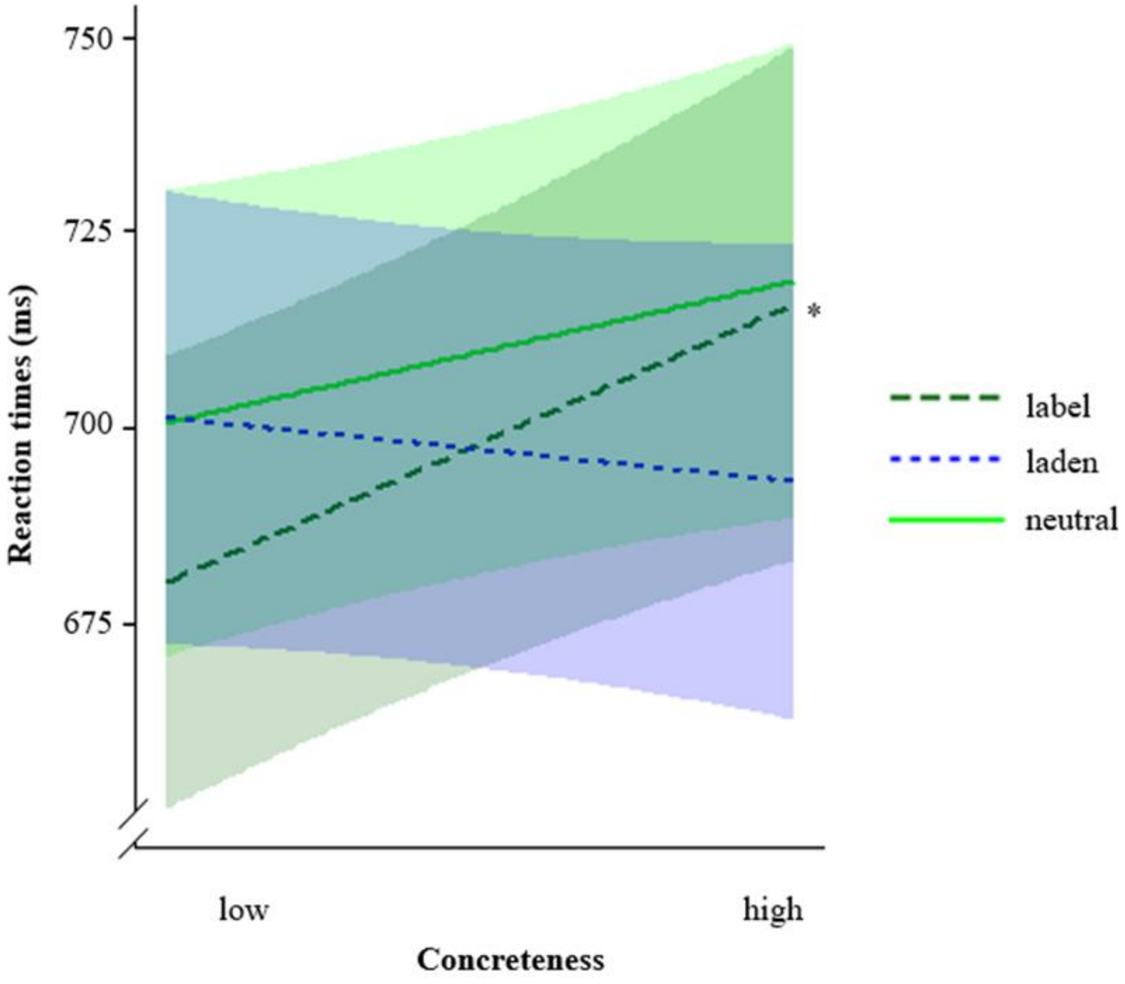

Note. Semitransparent ribbons reflect 90% confidence intervals. Label = emotion-label words; laden = emotion-laden words.

*p > .05

Figure B2

Slope estimates for the effect of Valence per Word Type on the reaction times in the lexical decision task in the LME analysis including the factor Signed Valence

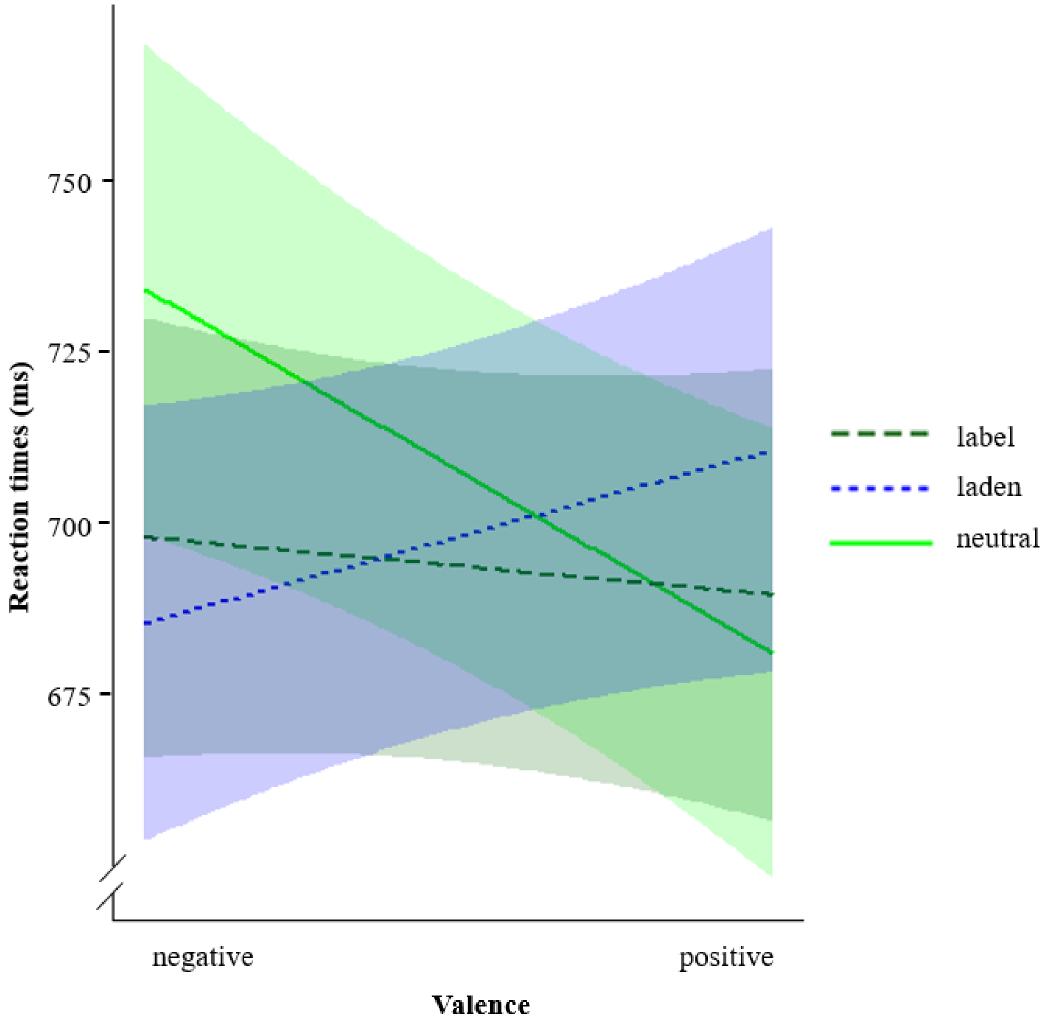

Note. Semitransparent ribbons reflect 90% confidence intervals. Label = emotion-label words; laden = emotion-laden words.

Table B1
Inferential statistics of the LME analysis on reaction time in the lexical decision task including the factor Signed Valence

Effect	β	*SE*	*df*	*t*/*F*[1]	*p*	
Word Type			2, 132.6	0.39	.675	
Empathy	-1.97	2.56	55.99	-0.77	.446	
Signed Valence	-6.59	2.87	5731.87	-2.30	.022	*
Arousal	1.35	2.05	5741.42	0.66	.510	
Concreteness	4.54	2.98	5772.46	1.52	.128	
Interoception	-7.57	3.77	5777.78	-2.01	.045	*
Word Type × Empathy			2, 5640.7	7.29	< .001	***
Resolution Word Type by Empathy						
Word Type for low Empathy			2, 156.6	0.10	.902	
Word Type for high Empathy			2, 179.6	2.11	.125	
Resolution Empathy by Word Type						
Planned Contrasts						
Empathy for Label vs. Neutral	-3.93	1.04	5650.36	-3.78	< .001	***
Empathy for Laden vs. Neutral	-2.39	1.02	5630.89	-2.35	.019	*
Empathy for Label vs. Laden	-1.54	1.03	5641.10	-1.50	.133	
Simple Slopes						
Empathy for Label	-5.90	2.56		-2.30	.025	*
Empathy for Laden	-4.36	2.56		-1.71	.094	
Empathy for Neutral	-1.96	2.56		-0.77	.446	
Word Type × Signed Valence			2, 1880.2	3.48	.031	*
Resolution Signed Valence by Word Type						
Planned Contrasts: Signed Valence for						
Label vs. Neutral	5.55	3.70	3495.04	1.50	.134	
Laden vs. Neutral	9.74	3.69	3852.43	2.64	.008	**
Label vs. Laden	-4.18	3.34	1167.67	-1.25	.210	
Simple Slopes						
Signed Valence for Label	-1.04	2.36		-0.44	.660	
Signed Valence for Laden	3.14	2.37		1.32	.186	
Signed Valence for Neutral	-6.59	2.87		-2.29	.022	*
Word Type × Arousal			2, 5721.1	0.36	.700	
Word Type × Concreteness			2, 5738.4	3.35	.035	*
Resolution Concreteness by Word Type						
Planned Contrasts: Concreteness for						
Label vs. Neutral	4.36	4.38	5733.28	1.00	.320	
Laden vs. Neutral	-6.57	4.07	5745.22	-1.62	.106	
Label vs. Laden	10.93	4.31	5716.64	2.54	.011	*
Simple Slopes						
Concreteness for Label	8.89	3.49		2.55	.011	*
Concreteness for Laden	-2.03	3.09		-0.66	.511	
Concreteness for Neutral	4.54	2.98		1.52	.128	
Word Type × Interoception			2, 5729.0	0.74	.478	

Note. LME = linear mixed effect, *SE* = standard error, *df* = degrees of freedom, label = emotion-label words, laden = emotion-laden words. The sign of the beta estimates shows the direction of effects.

Empathy effects for emotion-label, emotion-laden and neutral abstract words

[1]*t*-statistic for all effects except for main and interaction effects including Word Type. For effects including Word Type, *F*-statistic is reported and beta values are not available.
* $p < .05$, ** $p < .01$, *** $p < .001$

Appendix C

Analysis Including Gender

Method

An independent samples *t*-test revealed no difference in empathy scores between men and women, $t(50) = 1.21$, $p = .232$, $d = -0.37$. Nevertheless, we conducted an LME analysis on reaction times in the lexical decision task to test the effect of Gender. We defined a model including the categorical factor Word Type (within-subject, three levels: emotion-label, emotion-laden, neutral). To allow all three pairwise comparisons of the three word types, two contrast matrices were set up: one with neutral words as reference condition and one with emotion-label words as reference condition. We further included the categorical factor Gender (between-subject, two levels: male, female). The model also included the mean-centered, continuous factor Empathy as well as its interaction with Word Type and all their possible interactions as fixed effects. All the other fixed and random effects were modelled as in the main analysis reported in the manuscript. Specifically, the model was:

*Reaction times ~ Word Type*Empathy*Gender + Absolute Valence + Word Type:Absolute Valence + Arousal + Word Type:Arousal + Concreteness + Word Type:Concreteness + Interoception + Word Type:Interoception + (1|Participant) + (1|Word)*

We performed an outlier detection based on Cook's distance [1] using the R package influence.ME version 0.9-9 [2]. Values ranged from < 0.01 to 0.08 ($M = 0.02$, $SD = 0.02$). Thus, no subject's Cook's distance exceeded the cut-off of 1 originally suggested by Cook [1] nor the more conservative cut-off of ~ 0.16 suggested by Jayakumar and Sulthan [3] (based on simulations for our sample size and number of factors). We then applied the outlier criterion based on model criticism [4] by excluding trials with standardized residuals higher than 2.5 or lower than -2.5 ($n = 152$ data points). In total, 5803 data points were included.

Results

Empathy effects for emotion-label, emotion-laden and neutral abstract words

A detailed summary of the inferential statistics for this additional LME analysis on reaction times in the lexical decision task is reported in Table C1. This analysis revealed a pattern of results consistent with the main analysis reported in the manuscript. We found a significant Word Type × Empathy interaction, $p = .014$. Resolving this interaction via testing the effect of Word Type by Empathy, we found that the effect of Word Type was neither significant for participants with lower empathy, $p = .714$, nor for participants with higher empathy, $p = .388$. Resolving this interaction additionally via testing the effect of Empathy by Word Type, planned contrasts revealed a significantly stronger effect of Empathy for emotion-label than neutral words, $p = .006$, and for emotion-laden than neutral words, $p = .033$, while there was no significant difference between emotion-label and emotion-laden words, $p = .501$. However, the post-hoc simple slope analyses were not significant for any Word Type, all ps ≥ .176. Regarding the included rating-based covariates, there was a significant main effect of Interoception, p = .029, with higher interoception ratings leading to faster responses in the lexical decision task. All other main and interaction effects were not significant, all ps ≥ .080.

Concerning the effect of Gender specifically tested in this additional analysis, neither the main effect of Gender nor any other interaction involving Gender were significant, all ps ≥ .235.

Table C1

Inferential statistics of the LME analysis on reaction time in the lexical decision task including the factor Gender

Effect	β	*SE*	*df*	t/F^1	*p*	
Word Type			2, 146.9	0.13	.878	
Empathy	-0.76	3.06	53.67	-0.25	.804	
Gender	37.21	31.01	53.60	1.20	.235	
|Valence|	-1.11	3.69	5724.11	-0.30	.763	
Arousal	0.54	2.08	5742.27	0.26	.795	
Concreteness	4.20	2.99	5767.26	1.40	.160	
Interoception	-8.29	3.80	5772.35	-2.18	.029	*
Word Type × Empathy			2, 5625.2	4.29	.014	*
Resolution Word Type by Empathy						
Word Type for low Empathy			2, 170.3	0.34	.714	
Word Type for high Empathy			2, 219.4	0.95	.388	
Resolution Empathy by Word Type						
Planned Contrasts						
Empathy for Label vs. Neutral	-3.44	1.25	5645.01	-2.76	.006	**
Empathy for Laden vs. Neutral	-2.61	1.22	5633.08	-2.13	.033	*
Empathy for Label vs. Laden	-0.83	1.24	5631.08	-0.67	.501	
Simple Slopes						
Empathy for Label	-4.20	3.06		-1.37	.176	
Empathy for Laden	-3.37	3.05		-1.10	.275	
Empathy for Neutral	-0.76	3.06		-0.25	.804	
Word Type × Gender			2, 5625.5	1.40	.248	
Empathy × Gender			1, 48.0	0.23	.631	
Word Type × |Valence|			2, 5735.5	1.99	.137	
Word Type × Arousal			2, 5736.8	0.11	.895	
Word Type × Concreteness			2, 5735.0	2.53	.080	
Word Type × Interoception			2, 5720.6	0.80	.449	
Word Type × Empathy × Gender			2, 5621.5	0.05	.955	

Note. LME = linear mixed effect, |Valence| = absolute valence, *SE* = standard error, *df* = degrees of freedom, label = emotion-label words, laden = emotion-laden words. The sign of the beta estimates shows the direction of effects.

[1]*t*-statistic for all effects except for main and interaction effects including Word Type. For effects including Word Type, *F*-statistic is reported and beta values are not available.

* $p < .05$, ** $p < .01$

Appendix D

Analysis Excluding Emotional Covariates

Method

We conducted an additional LME analysis on reaction times in the lexical decision task without modelling valence, arousal and interoception as covariates to test whether their inclusion in the main LME analysis (as reported in the manuscript) prevented the main effect of emotionality from becoming significant. The model was the same as reported in the manuscript, but including only Concreteness as covariate:

*Reaction times ~ Word Type*Empathy + Concreteness + Word Type:Concreteness + (1|Participant) + (1|Word)*

We performed an outlier detection based on Cook's distance [1] using the R package influence.ME version 0.9-9 [2]. Values ranged from < 0.01 to 0.08 (M = 0.01, SD = 0.02). Thus, none of the Cook's distance values exceed the cut-off of 1 originally suggested by Cook [1] nor the more conservative cut-off of ~ 0.16 suggested by Jayakumar and Sulthan [3] (based on simulations for our sample size and number of factors). We then applied an outlier criterion based on model criticism [4] by excluding trials with standardized residuals higher than 2.5 or lower than -2.5 (n = 151 data points). After exclusions, a total of 5804 data points were included into the LME analysis on the reaction times in the lexical decision task.

Results

The LME analysis on reaction times in the lexical decision task only including the Concreteness covariate revealed a significant Word Type × Empathy interaction, $p < .001$, displayed in Figure D1. The resolution of the interaction followed the same procedure as described in the manuscript and revealed corresponding results for the resolution by Word Type and simple slope analyses as displayed in Table D1. The resolution by Empathy revealed a deviation from the results of the main analysis reported in the manuscript, as the effect of Word Type was significant for participants with a higher Empathy, $p = .023$. Planned

contrasts revealed significantly faster reaction times for emotion-label than neutral words in participants with higher Empathy, p =.006, while reaction times for emotion-laden words did not differ significantly from those for neutral, p = .101, or emotion-label words, p = .262.

Regarding the included rating-based covariate of Concreteness, the analysis revealed a significant Word Type × Concreteness interaction, p = .040, displayed in Figure D2. Resolving this interaction via testing the effect of Concreteness by Word Type, planned contrasts revealed a significantly stronger effect of Concreteness for emotion-label than emotion-laden words, p = .015, while there was neither a significant difference between emotion-label and neutral words, p = .422, nor emotion-laden and neutral words, p = .083. Post-hoc simple slope analyses revealed that higher concreteness ratings led to significantly faster reaction times in response to emotion-label words, p = .015, while no significant effect was observed for emotion-laden, p = .508, nor neutral words, p = .092. All other main and interaction effects were not significant, all ps ≥ .092. For inferential statistics, see Table D1.

Empathy effects for emotion-label, emotion-laden and neutral abstract words

Figure D1

Slope estimates for the effect of Empathy per Word Type on the reaction times in the lexical decision task in the LME analysis including only the Concreteness covariate

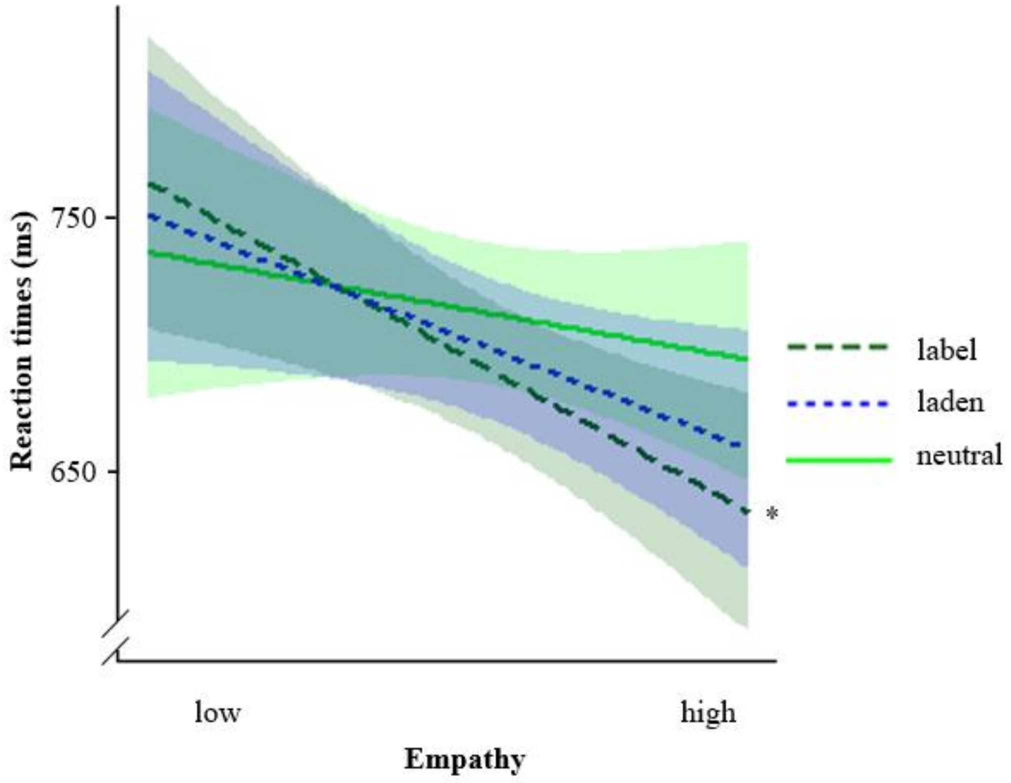

Note. Semitransparent ribbons reflect 90% confidence intervals. Label = emotion-label words; laden = emotion-laden words.
*p < .05

Figure D2

Slope estimates for the effect of Concreteness per Word Type on the reaction times in the lexical decision task in the LME analysis including only the Concreteness covariate

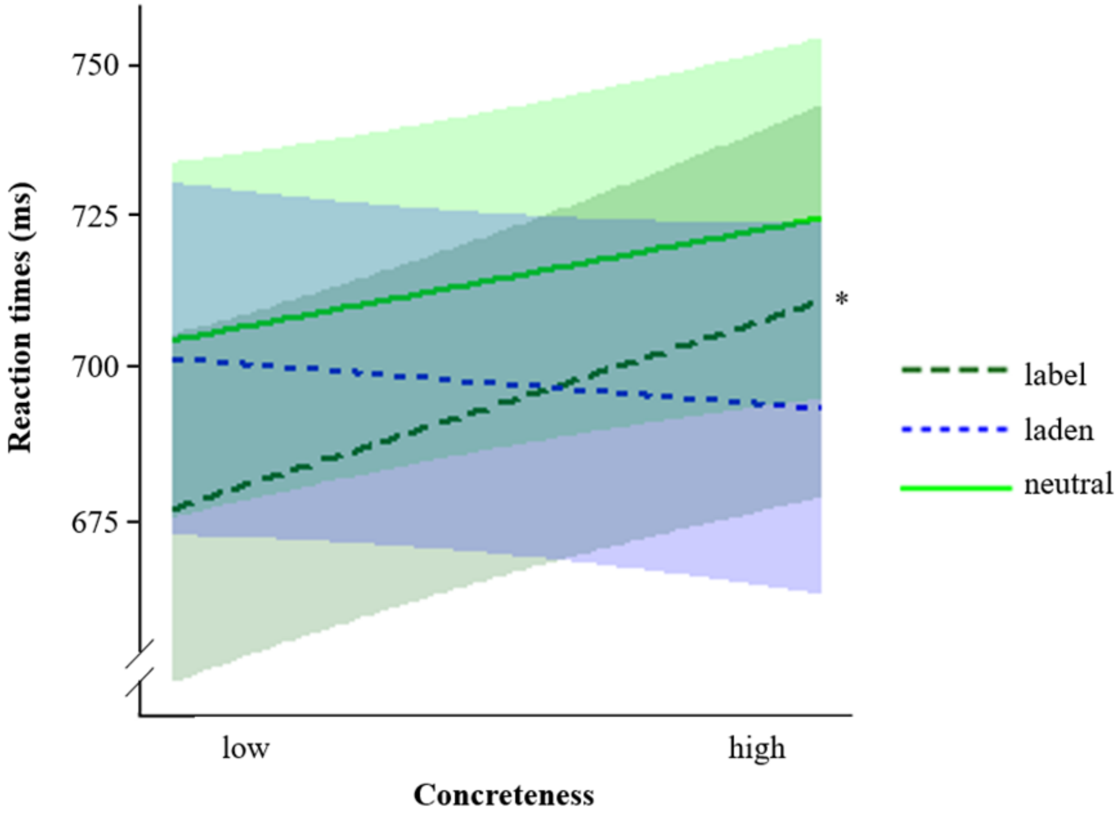

Note. Semitransparent ribbons reflect 90% confidence intervals. Label = emotion-label words; laden = emotion-laden words.
*$p < .05$

Table D1

Inferential statistics of the LME analysis on reaction time in the lexical decision task including only the Concreteness covariate

Effect	β	*SE*	*df*	*t*/*F*[1]	*p*	
Word Type			2, 115.8	1.11	.333	
Empathy	-2.01	2.54	55.47	-0.79	.433	
Concreteness	4.98	2.96	5783.07	1.69	.092	
Word Type × Empathy			2, 5631.4	8.65	< .001	***
Resolution Word Type by Empathy						
Word Type for low Empathy			2, 145.6	< 0.01	.998	
Word Type for high Empathy			2, 148.4	3.89	.023	*
Planned Contrasts						
Label vs. Neutral	-44.53	16.08	150.49	-2.77	.006	**
Laden vs. Neutral	-26.35	15.96	146.14	-1.65	.101	
Label vs. Laden	-18.18	16.13	148.51	-1.13	.262	
Resolution Empathy by Word Type						
Planned Contrasts						
Empathy for Label vs. Neutral	-4.15	1.00	5632.27	-4.14	< .001	***
Empathy for Laden vs. Neutral	-2.36	1.00	5630.71	-2.37	.018	*
Empathy for Label vs. Laden	-1.78	1.01	5631.12	-1.77	.076	
Simple Slopes						
Empathy for Label	-6.16	2.54		-2.42	.019	*
Empathy for Laden	-4.37	2.54		-1.72	.091	
Empathy for Neutral	-2.01	2.54		-0.79	.433	
Word Type × Concreteness			2, 5744.3	3.23	.040	*
Resolution Concreteness by Word Type						
Planned Contrasts: Concreteness for						
Label vs. Neutral	3.50	4.36	5737.51	0.80	.422	
Laden vs. Neutral	-7.03	4.06	5754.52	-1.73	.083	
Label vs. Laden	10.53	4.31	5720.93	2.44	.015	*
Simple Slopes						
Concreteness for Label	8.49	3.48		2.44	.015	*
Concreteness for Laden	-2.04	3.09		-0.66	.508	
Concreteness for Neutral	4.98	2.96		1.69	.092	

Note. LME = linear mixed effect, *SE* = standard error, *df* = degrees of freedom, label = emotion-label words, laden = emotion-laden words. The sign of the beta estimates shows the direction of effects.

[1]*t*-statistic for all effects except for main and interaction effects including Word Type. For effects including Word Type, *F*-statistic is reported and beta values are not available.

* $p < .05$, ** $p < .01$, *** $p < .001$

References

1 Cook, R. D. Detection of Influential Observation in Linear Regression. *Technometrics* **19**, 15-18, doi:10.1080/00401706.1977.10489493 (1977).

2 Nieuwenhuis, R., Te Grotenhuis, M. & Pelzer, B. influence.ME: Tools for Detecting Influential Data in Mixed Effects Models. *R Journal* **4**, 38-47, doi:10.31235/osf.io/a5w4u (2012).

3 Jayakumar, G. S. D. S. & Sulthan, A. Exact Distribution of Cook S Distance and Identification of Influential Observations. *Hacettepe Journal of Mathematics and Statistics* **44**, 1-1, doi:10.15672/hjms.201487459 (2014).

4 Baayen, R. H. & Milin, P. Analyzing Reaction Times. *International Journal of Psychological Research* **3**, 12-28 (2010).
